# A comparison of ICD-11 and DSM-5 criteria of PTSD among Chinese trauma-exposed adolescent samples

**DOI:** 10.3389/fpsyt.2023.1186138

**Published:** 2023-06-13

**Authors:** Li Wang, Ruojiao Fang, Chen Chen, Chengqi Cao

**Affiliations:** ^1^Laboratory for Traumatic Stress Studies, CAS Key Laboratory of Mental Health, Institute of Psychology, Chinese Academy of Sciences, Beijing, China; ^2^Department of Psychology, University of Chinese Academy of Sciences, Beijing, China

**Keywords:** DSM-5, ICD-11, post traumatic stress disorder, adolescent, descriptive survey study

## Abstract

This study aimed at comparing the prevalence and comorbidity differences of PTSD according to ICD-11 and DSM-5 definitions across two Chinese adolescent trauma-exposed samples. A total of 1,201 students exposed to earthquake and 559 students from vocational schools exposed to potentially traumatic events were included in this study. The PTSD Checklist for DSM-5 was used to measure PTSD symptoms. The MDD and GAD subscales of the Revised Children’s Anxiety and Depression Scale were used to measure major depression disorder (MDD) and generalized anxiety disorder (GAD) symptoms. No significant PTSD prevalence differences between ICD-11 and DSM-5 were found across the two samples. The differences regarding comorbidities between ICD-11 and DSM-5 definitions were not significant among these two samples. The results revealed that the ICD-11 and DSM-5 provided similar prevalence of PTSD and comorbidity rates with MDD and GAD in Chinese trauma-exposed adolescent samples. This study contributes to the current understanding of the similarities and differences using different PTSD criteria and informs the organization and application of these two globally applied PTSD criteria.

## Introduction

Exposure to traumatic events is widespread among adolescents. According to a national survey of the United States, a majority (60.0%) of adolescents aged 13 to 18 experienced one or more traumatic events (1). Posttraumatic stress disorder (PTSD) is one of the most prevalent mental problems among trauma-exposed adolescents [e.g., (2)]. The diagnosis of PTSD is commonly based on the International Classification of Diseases (ICD) by the World Health Organization (WHO) and the Diagnostic and Statistical Manual of Mental Disorders (DSM) by the American Psychiatric Association (APA) worldwide. Nevertheless, the definition of PTSD in the recent version of the ICD (ICD-11; (3)) differs markedly from the latest DSM (DSM-5; (4)). More concretely, the DSM-5 defines PTSD in a broad way and includes twenty symptoms. The DSM-5 criteria require the presence of at least one out of five intrusion symptoms, one out of two avoidance symptoms, two out of seven negative alterations in cognitions and mood symptoms, and two out of six alterations in arousal and reactivity symptoms to diagnose PTSD. In contrast, the ICD-11 defines PTSD in a narrow way by eliminating “non-specific” PTSD symptoms. Therefore, the ICD-11 includes six “specific” PTSD symptoms and requires at least one of two intrusion symptoms, one of two avoidance symptoms, and one of two sense of threat symptoms to diagnose PTSD (5). By using a narrower and briefer set of symptoms, ICD-11 aims to ease diagnostic application, reduce psychiatric comorbidity, and include more symptomatic individuals. Taken together, these two criteria adopt vastly different conceptualizations of PTSD, as the DSM-5 defines PTSD as a multifaceted and complex syndrome while the ICD-11 regards PTSD as a more specific post-traumatic psychological response. The different definitions of PTSD would affect our understanding of the traumatic stress response.

In consideration of the striking distinction between ICD-11 and DSM-5 in the diagnostic conceptualization of PTSD, a number of studies focused on comparing differences between these two divergent diagnostic systems. In general, most of the studies were conducted among adults and almost exclusively showed that the ICD-11 criteria provide a lower prevalence than the DSM-5 [e.g., (6–9)]. Studies further evaluating the comorbidity differences between these two criteria of PTSD demonstrated mixed results. Several studies showed that using the ICD-11 and DSM-5 criteria provided equivalent comorbidity rates between PTSD and other mental disorders such as major depression disorder (MDD) [e.g., (10, 11)] and generalized anxiety disorder (GAD) (11). However, other studies showed that the DSM-5 could significantly increase the co-occurrence rate [e.g., (12, 13)]. In addition, Shevlin et al. (9) reported a lower co-occurring rate with MDD and GAD for DSM-5 criteria.

Only a few studies were conducted among youths, and mixed results were reported. Sachser et al. (14) showed a lower prevalence of ICD-11 than DSM-5 among youths who experienced potentially traumatic events. However, other studies found that the ICD-11 and DSM-5 provided equivalent prevalence of PTSD among children and adolescents, including two samples of survivors of hurricanes (15), survivors of the terrorist attack (7) and foster children (16). Regarding PTSD comorbidity differences, only two studies demonstrated that the DSM-5 criteria yielded higher comorbidity with MDD and GAD compared with ICD-11 (7, 16).

As previously mentioned, compared to a large number of studies among adults, there are only a handful of studies that compare the prevalence of ICD-11 and DSM-5 PTSD among youths. Moreover, the previous studies were all conducted in the Western world. As culture has important effects on the prevalence and presentation of PTSD [e.g., (17, 18)], studies elucidating differences between the ICD-11 and DSM-5 criteria for PTSD within non-Western samples would be informative for the utility of these two globally-used systems. The current study first evaluated the agreement between ICD-11 and DSM-5 criteria in two Chinese adolescent samples: the earthquake-exposed sample and the vocational school sample. Subsequently, this study further examined the coexisting prevalence of PTSD and MDD as well as GAD.

## Materials and methods

### Participants and procedure

#### Earthquake-exposed sample

The current study was a cross-sectional study conducted in two junior high schools in Beichuan County. The 2008 Wenchuan Earthquake almost destroyed the Beichuan County, leaving more than 15,000 people dead there. The current study was conducted approximately 6.5 years after the earthquake. Investigators, including trained research assistants and school teachers, introduced the aim of this survey in details, and then administered self-reported questionnaires to the participants in class groups. Informed consent was obtained from both the participants and their guardians. This study was approved by the Institutional Review Board of our Institute.

A total of 1,206 students personally experiencing the disaster were included in this study. As five participants were excluded from this study for missing more than 20 % items of questionnaires, the final sample was 1,201 students (53.2% girls and 46.0% boys) with ages ranging from 13 to 17 years (mean = 14.3 years, *SD* = 0.8). In terms of ethnicity, 769 participants (64.0%) self-reported as Qiang, 387 (32.2%) were Han, and 27 (2.2%) were other ethnicities in China.

#### Vocational school sample

The current study was a cross-sectional study conducted in a vocational school and an approved school located in Beijing, and an approved school located in Changsha. The current study was conducted by class groups with the monitoring of trained research assistants and school teachers from September to October 2014. After introducing the aim of this study, investigators obtained informed consent and administered self-reported questionnaires to the participants. This study was approved by the Institutional Review Board of our Institute.

The Part I of the University of California at Los Angeles Posttraumatic Stress Disorder Reaction Index (PTSD-RI) (19) was applied to screen potentially traumatic events and identify the index traumatic event for assessing PTSD symptoms. Among the initial sample of 1,023 students, 559 students (242 girls and 314 boys) successfully appointed an index traumatic event were included in this study. The age of this sample ranged from 12 to 18 (mean = 15.8 years, *SD* = 1.3). Regarding ethnicity, the majority of participants (94.5%) were Chinese Han ethnicity. Participants reported being exposed to 2.5 traumatic events on average (range: 1–11, *SD* = 1.6). The top three index traumatic events that the participants reported were “Seeing someone in your town being beaten up, shot at or killed” (19.5%), “Being hit, punched, or kicked very hard at home” (18.2%), and “Hearing about the violent death or serious injury of a loved one” (14.0%) [Detailed information about the index traumatic event please referred to (20)].

### Measures

The PTSD Checklist for DSM-5 (PCL-5) (21) was used to measure PTSD symptoms. The PCL-5 is a 20-item self-reported measure rated on a five-point Likert-scale (0 = *not at all* to 4 = *extremely*) to capture PTSD symptoms including intrusion symptoms (e.g., intrusive thoughts), avoidance symptoms (e.g., avoidance of thoughts of trauma), negative alterations in cognitions and mood symptoms (e.g., trauma-related amnesia), and alterations in arousal and reactivity symptoms (e.g., irritability or aggressive behavior) in the past month. The PCL-5 was answered referring to the index traumatic event selected in the PTSD-RI among the vocational school sample and the Wenchuan earthquake among the earthquake-exposed sample, respectively. The symptom scored two or greater indicated the existence of this symptom. The diagnoses of the DSM-5 and ICD-11 PTSD cases were based on criteria mentioned earlier (please refer to the first paragraph in the introduction). The Chinese version of PCL-5 has been validated among Chinese trauma-exposed adolescents [e.g., (22, 23)]. Cronbach’s alphas for six items of the PCL-5 for the ICD-11 and twenty items for the DSM-5 were.82 and 0.94 among the earthquake-exposed sample, and 0.81 and.94 among the vocational school sample, respectively.

The MDD and GAD subscales of the Revised Children’s Anxiety and Depression Scale (RCADS) (21) were used to measure MDD (e.g., having trouble sleeping) and GAD symptoms (e.g., worrying something bad will happen to self). The MDD and GAD subscales are 10-item and 6-item scales rated from 0 (*never*) to 3 (*always*) to reflect the frequency of a particular symptom during the past two weeks, respectively. A cutoff score of at least 11 has been recommended to identify possible MDD cases, while a cutoff score of at least seven has been recommended to identify possible GAD cases (24). Cronbach’s alphas for the MDD subscales and the GAD subscales were 0.87 and 0.88 among the earthquake-exposed sample, and 0.79 and.79 among the vocational school sample, respectively.

### Statistical analyses

The SPSS 20.0 was used for data analysis. First, prevalence rates with 95% confidence intervals of prevalence based on the DSM-5 and ICD-11 criteria among these two samples were estimated. The differences across diagnostic systems were statistically compared with the Z-test. Second, diagnostic concordance between these two PTSD criteria was evaluated with the Cohen’s Kappa (*κ*) (0.61–0.80: substantial agreement and ≥ 0.80: almost perfect agreement) (25). Third, differences in comorbidity rates among DSM-5 and ICD-11 PTSD groups were assessed with the Z-test.

## Results

### Prevalence of PTSD

The DSM-5 and ICD-11 PTSD prevalences were 5.3% (64 participants; 95% *CI* = 4.1–6.7%) and 4.4% (53 participants; 95% *CI* = 3.3–5.6%) among the earthquake-exposed sample, respectively. The difference between the rates of these two diagnostic systems was not significant within this sample (*Z* = 1.03, *p* = 0.152). Among the vocational school sample, the rate of PTSD using DSM-5 was 12.3% (69 participants; 95% *CI* = 9.7–15.0%) which was exactly the same as using ICD-11. The difference between the rates of these two diagnostic systems was not significant within this sample (*Z* = 0, *p* > 0.1).

### Diagnostic agreement

Table 1 demonstrated diagnostic concordance and discordance between ICD-11 and DSM-5. Estimates of Cohen’s kappa between these two criteria among the earthquake-exposed sample and the vocational school sample were 0.79 and 0.70, respectively, indicating substantial agreement. For the 70 probable PTSD cases diagnosed with either ICD-11 or DSM-5, there was a diagnosis discordance rate of 32.9% (23 participants) among the earthquake-exposed sample. For the 87 probable PTSD cases diagnosed with either ICD-11 or DSM-5, there was a diagnosis discordance rate of 41.4% (36 participants) among the vocational school sample.

**Table 1:** Patterns of agreement and disagreement between ICD-11 and DSM-5 PTSD diagnoses in these two samples.

		DSM-5 Diagnosis		
		Negative	Positive	Total
ICD-11 Diagnosis	Earthquake-exposed sample			
	Negative	1,131 (94.2)	17 (1.4)	1,148 (95.6)
	Positive	6 (0.5)	47 (3.9)	53 (4.4)
	Total	1,137 (94.7)	64 (5.3)	1,201 (100.0)
	Vocational school sample			
	Negative	472 (84.4)	18 (3.2)	490 (87.7)
	Positive	18 (3.2)	51 (9.1)	69 (12.3)
	Total	490 (87.7)	69 (12.3)	559 (100.0)

The numbers in front of each cell are the number of participants and the numbers in the parentheses are the percentage of total.

### Comorbidity with MDD and GAD

Table 2 demonstrated the co-occurrence rate with MDD and GAD among PTSD cases based on ICD-11 and DSM-5. Among the earthquake-exposed sample, the co-occurrence rates were slightly lower according to ICD-11 than DSM-5. Among the vocational school sample, the co-occurrence rate with MDD among ICD-11 PTSD cases was slightly lower than the DSM-5 while the co-occurrence rates with GAD were slightly higher. However, the differences regarding comorbidities with MDD and GAD based on ICD-11 and DSM-5 criteria in these two samples were not significant (all *p* values >0.05).

**Table 2:** Co-occurrence of depression and anxiety for cases meeting criteria for the DSM-5 and ICD-11 PTSD diagnosis.

	Co-occurrence with MDD (%)			Co-occurrence with GAD (%)		
	*n*	%	95% CI	*n*	%	95% CI
Earthquake-exposed sample						
DSM-5 PTSD (*n* = 64)	53	82.8	73.2–91.5	54	84.4	75.0–92.9
ICD-11 PTSD (*n* = 53)	41	77.4	65.5–88.3	44	83.0	73.0–92.7
Vocational school sample						
DSM-5 PTSD (*n* = 69)	41	59.4	47.7–71.1	57	82.6	73.1–91.3
ICD-11 PTSD (*n* = 69)	40	58.0	46.2–69.1	61	88.4	80.0–95.5

PTSD, Posttraumatic stress disorder; MDD, major depression disorder; GAD, generalized anxiety disorder.

95% CI: 95% confidence intervals for prevalence rates.

## Discussion

The current study is the first to evaluate the prevalence and comorbidity based on ICD-11 and DSM-5 PTSD criteria among two samples of Chinese youth. The results showed that the PTSD prevalence based on DSM-5 was not significantly different from ICD-11 across two different trauma samples. A substantial agreement was found between these two criteria for PTSD. Additionally, the co-occurrence rate of PTSD with MDD and GAD was not significantly different between the ICD-11 and DSM-5 systems in these two samples.

The current study found the DSM-5 PTSD prevalence was not significantly different from the ICD-11. These results are consistent with prior studies that found a similar prevalence rate of PTSD across the two diagnostic systems [e.g., (7, 15, 16)]. The two diagnostic systems tend to have comparable clinical utility as yielding quantitatively similar proportions of PTSD cases. Moreover, this study found substantial agreement between these two criteria. These results are notable considering that the ICD-11 criteria are substantially briefer than the DSM-5 (6 symptoms for ICD-11 verse 20 symptoms for DSM-5). The briefer ICD-11 could greatly simplify the diagnosis and reduce the assessment burden on health care providers (5).

The agreement to a substantial degree is of particular note as the ICD-11 and DSM-5 criteria assess essentially the same disorder. Specifically, more than 30 % of the probable PTSD cases met only one criterion across these two samples in this study. The low agreement found in this study was congruent with previous studies showing only partial overlap to detect PTSD across different diagnostic systems [e.g., (15)]. These findings suggest that using different criteria for PTSD may have a qualitative impact on diagnostic decisions. In consideration of the worldwide use of these two systems (26), the divergences will be challenging for researchers and clinical psychologists in the area of trauma psychology. Further efforts should be made to minimize the inconsistencies between the ICD-11 and DSM-5 criteria for PTSD.

The primary goal of the ICD-11 revision to “narrow” PTSD was to decrease co-occurring with other common mental disorders. However, the results of this study together with previous studies among youths (7, 16) showed no evidence to decrease co-occurrence with GAD and MDD of ICD-11 PTSD, indicating that removing ‘non-specific symptoms’ may not decrease co-existing rate. Actually, comorbidity is a common phenomenon instead of an anomaly in mental disorders and is extensively considered to reflect the fact that a broad range of symptoms share a common neural basis (27). Therefore, the notion that the exceedingly common comorbidity is problematic and needs to be fixed may be questionable in itself.

Noticeably, the comparisons between ICD-11 and DSM-5 PTSD criteria were based primarily on non-western adult studies. It is crucial to evaluate the impact of using different criteria on PTSD prevalence and comorbidity among Chinese trauma-exposed youth. The current study may have significance for practice and research. In some adult studies, the rate of DSM-5 PTSD was significantly higher than that of ICD-11 [e.g., (12, 28, 29)], and researchers argued that the intrusion cluster of ICD-11 PTSD might be expanded to include the re-experiencing symptom to increase the prevalence of PTSD [e.g., (12)]. However, the comparable PTSD prevalence across two samples in this study indicates that such expansion may not be necessary. Moreover, the results showed that these two systems might identify different individuals. This discrepancy across these two systems raises challenges for researchers to identify etiological factors for PTSD. In addition, what calls for special attention is that research outcomes using the DSM system may not generalize to populations using the ICD criteria as the two systems may depend on different study populations, and vice versa.

There are several limitations of this study. At first, we used the PCL-5 to measure ICD-11 PTSD symptoms, which provided less accuracy (e.g., the ICD-11 defines intrusions as re-experiencing the traumatic events in the present that did not capture by PCL-5) compared with a standardized measure of ICD-11 PTSD symptoms, such as the International Trauma Questionnaire (30). Second, we did not assess the complex PTSD in ICD-11 and the functional impairment required in PTSD diagnoses in this study. Further studies using specialized ICD-11 measurements together with the assessment of the complex PTSD symptoms and functional impairment are needed. Third, we used self-reported measures to assess the symptoms of participants. Future studies with interview-based assessments should be conducted. Finally, the relatively low prevalence of PTSD in current studies limited the possibility to explore comorbidity differences between “unique” ICD-11 and DSM-5 PTSD cases.

Notwithstanding these limitations, this study is the first to compare ICD-11 and DSM-5 criteria for PTSD across two Chinese adolescent trauma-exposed samples. The results revealed that the ICD-11 and DSM-5 provided similar prevalence of PTSD and comorbidity rates with MDD and GAD. This study contributes to the current understanding of the similarities and differences using different PTSD criteria and informs the organization and application of these two globally applied PTSD criteria.

## Data availability statement

The raw data supporting the conclusions of this article will be made available by the authors, without undue reservation.

## Ethics statement

The studies involving human participants were reviewed and approved by the Institutional Review Board of Institute of Psychology, Chinese Academy of Science. Written informed consent to participate in this study was provided by the participants’ legal guardian/next of kin.

## Author contributions

LW conceptualized the idea for this study, edited the manuscript, and secured funding. RF was responsible for data collection. CCa conducted the analyses and wrote the first draft of the manuscript. CCh assisted with the analyses. All authors contributed to the article and approved the submitted version.

## Funding

This study was partially supported by the National Natural Science Foundation of China (No. U21A20364 and No. 31971020), the Key Project of the National Social Science Foundation of China (No. 20ZDA079), the Key Project of Research Base of Humanities and Social Sciences of Ministry of Education (No. 16JJD190006), the Scientific Foundation of Institute of Psychology, Chinese Academy of Sciences (No. E2CX4115CX), and the Scientific Foundation of Institute of Psychology, Chinese Academy of Sciences (No. E1CX161005).

## Conflict of interest

The authors declare that the research was conducted in the absence of any commercial or financial relationships that could be construed as a potential conflict of interest.

## Publisher’s note

All claims expressed in this article are solely those of the authors and do not necessarily represent those of their affiliated organizations, or those of the publisher, the editors and the reviewers. Any product that may be evaluated in this article, or claim that may be made by its manufacturer, is not guaranteed or endorsed by the publisher.
